# Retraction Note: Identification of a novel Na^+^-coupled Fe^3+^-citrate transport system, distinct from mammalian INDY, for uptake of citrate in mammalian cells

**DOI:** 10.1038/s41598-018-28645-x

**Published:** 2018-07-17

**Authors:** Jiro Ogura, Ellappan Babu, Seiji Miyauchi, Sabarish Ramachandran, Elizebeta Nemeth, Yangzom D. Bhutia, Vadivel Ganapathy

**Affiliations:** 1 Department of Cell Biology and Biochemistry, Texas Tech University Health Sciences Center, Lubbock, TX 79430 USA; 2 Department of Pharmaceutics, Toho University, Funabashi, Chiba 274-8510 Japan; 3 Department of Medicine and Center for Iron Disorders, University of California at Los Angeles, Los Angeles, CA 90095 USA

Retraction of: *Scientific Reports* 10.1038/s41598-018-20620-w, published online 06 February 2018

The authors are retracting this Article.

We were alerted to the possibility that the results described in the Article may be artefactual for the following reasons. Radiolabeled citrate binds to plastic culture dishes in the presence of ferric chloride. In the presence of excess unlabelled citrate and other iron chelators, this binding to plastic dishes may be inhibited, thus mimicking substrate selectivity and saturation kinetics.

We have now performed additional control experiments using culture plates without cells present, and obtained the same results as those described in the Article. The binding of ferric citrate to the plastic dishes showed sodium-dependence because N-methyl-D-glucamine chloride that we routinely use to substitute for NaCl to study sodium-dependence of a transport system interfered with the process.

As such, we are unable to support the conclusions regarding a new sodium-dependent ferric-citrate transporter in the mammalian cells.

All authors agree to the retraction of the Article.

